# Assessing the Understanding of Primary School Teachers in the Rural Communities of Limpopo Province, South Africa—Are We Trauma Informed?

**DOI:** 10.3390/children12010054

**Published:** 2025-01-02

**Authors:** Muimeleli Munyadziwa, Lufuno Makhado, Angelina Maphula

**Affiliations:** 1 Faculty of Health Sciences, Department of Public Health, University of Venda, Thohoyandou 0950, South Africa; lufuno.makhado@univen.ac.za; 2 Faculty of Health Sciences, Department of Psychology, University of Venda, Thohoyandou 0950, South Africa; angelina.maphula@univen.ac.za

**Keywords:** child trauma, trauma-exposed learners, understanding, primary schools, primary teachers

## Abstract

Background/Objectives: To effectively support children’s learning and well-being, primary educators must thoroughly understand child trauma. Being ‘trauma informed’ means recognizing the impact of trauma and responding supportively, which can help mitigate its adverse effects on learners. This study explored the understanding of childhood trauma among primary school teachers in Limpopo province, focusing on the circuits of Mvudi and Dzindi due to their high prevalence of childhood traumatic experiences. Methods: An exploratory descriptive and contextual design was used in this study. Data were collected through semi-structured interviews with 26 teachers, utilizing total population sampling for schools and purposive sampling for respondents. Results: The findings revealed that teachers typically define trauma in terms of domestic issues and abuse and identify symptoms in learners’ behavior, emotions, and academic performance. Teachers employ strategies to assist affected learners, such as building rapport and involving social workers or school authorities when needed. Conclusions: This study highlighted teachers’ critical role in identifying and assisting traumatized children and underlined the necessity for effective training programs and school-based support systems. A comprehensive strategy is necessary, including advocacy for equitable support services and standardized training on trauma-informed practices.

## 1. Introduction

The widespread problem of childhood trauma has significant effects on learners’ educational experiences and performance [1]. Trauma is broadly defined as events that exceed a child’s ability to cope, and it can take many different forms, including abuse, neglect, or witnessing violence [2]. According to the Substance Abuse and Mental Health Services Administration (SAMHSA), trauma is further described as an event, series of events, or set of circumstances that is experienced by an individual as physically or emotionally harmful or threatening and has lasting adverse effects on the individual’s functioning and well-being [2]. Learners’ social interactions, emotional control, and cognitive development can all be significantly impacted by these experiences, which frequently result in behavioral problems in the classroom and difficulties in school [3,4], as well as neurological and social development issues [5], which, at a later stage, can also lead to difficulties in internal regulation [6]. According to the World Health Organization (WHO), up to 1 billion children between the ages of two (2) and seventeen (17) are thought to have experienced sexual, emotional, and physical abuse or abandonment in the previous year worldwide [7]. In our research area, which was the Vhembe district, there were 200 reported cases of sexual assault in 2018 [8]. According to Thohoyandou Victim Empowerment (TVEP), there are children under thirteen (13) years of age account for about 60% of the victims of sexual assault [8]. According to Polity, the statistics on child abuse in the province of Limpopo rose rather than fell in the past two years, with attempts at sexual offences increasing by 31.7%, rape cases by 4.5%, and sexual assaults by 33.3%. In terms of reporting the number of rape cases, the Thohoyandou Police Station was among the top 30 stations [9]. These statistics show a waging battle against children that need immediate attention and drastic measures to combat this issue.

Since children spend significant time at school, teachers are vital in identifying and helping traumatized learners because they are frontline educators [10,11]. Trauma-sensitive approaches designed for schools [12,13,14] have the capacity to identify indicators of trauma, offer emotional support, and coordinate suitable interventions, which is essential for reducing the adverse consequences of trauma and fostering learners’ adaptability [15,16].

Nonetheless, studies indicated that many educators believe they are not well-equipped to deal with trauma [17], not having received enough training or resources to meet the complex needs of traumatized learners, which causes adverse outcomes for the learners. The outcomes may include memory, learning, social interaction, and emotional control issues. These issues may result in behavioral and relational problems in the classroom [18,19].

In the context of South Africa, where this study was conducted, the educational system faces significant challenges related to learners’ mental health and well-being [20]. South African schools, particularly in rural areas such as the Vhembe district, are often under-resourced and may lack the necessary infrastructure and professional support services to address trauma effectively [21]. Mental health care and trauma-informed approaches are still developing within the school system, and many teachers report insufficient training in handling the emotional and psychological needs of students. South Africa’s Department of Basic Education has tried incorporating mental health services into schools, especially by integrating psycho-social support programs. Still, these services are not universally available or adequately funded [22,23]. In some regions, such as Vhembe, where a high incidence of sexual violence and abuse is reported [8], school-based counselling and mental health resources are limited. Teachers, therefore, often serve as the primary point of contact for identifying and supporting traumatized students.

This study sought to investigate primary school teachers’ understanding of emotional and psychological trauma in learners in Limpopo Province. We specifically aimed to identify the indicators that teachers use to recognize trauma, the strategies they implement to support affected learners, and the availability and effectiveness of trauma-related resources in schools. This research also explored the gaps in teacher training and the need for additional support to create safer and more inclusive learning environments.

Comprehending and tackling these obstacles is imperative for enhancing academic achievements for traumatized pupils and cultivating a more empathetic and adaptable educational framework. Schools can improve all learners’ well-being and academic achievement by meeting their diverse needs and providing teachers with adequate knowledge, skills, and support systems [19].

## 2. Materials and Methods

### 2.1. Research Ethics Board Approval Information

This study was carried out following the Declaration of Helsinki and received approval from the Human and Clinical Trial Research Ethics Committee at the University of Venda (FHS/24/PH/46, approved on 18 November 2024). The Department of Basic Education granted permission to conduct research in the Limpopo Province and by the selected schools’ districts, circuits, and principals. Before the interviews, all primary school teachers provided voluntary written informed consent. They were fully briefed on the potential risks and benefits of this study. Confidentiality was maintained throughout the research process. Participation was voluntary, and participants were free to withdraw from the study at any time without any negative consequences.

### 2.2. Study Design

An exploratory descriptive and contextual design was used in this study. The researchers outlined and investigated primary school teachers’ understanding when assisting learners who have experienced trauma. An exploratory descriptive design is an approach that can help summarize and comprehend an area of interest [24,25]. This design was chosen because it allows for an in-depth exploration of a relatively under-researched topic, capturing teachers’ perceptions, practices, and strategies in a specific educational context. This study’s exploratory nature helped uncover teachers’ knowledge and attitudes toward trauma, while the descriptive component focused on documenting how these teachers define trauma, identify it in students, and respond to it. These areas were explored through structured interview questions designed to gather detailed responses on their experiences and practices. The contextual aspect ensured that this study specifically focused on the unique circumstances of the Vhembe District, considering the local educational environment and socio-cultural factors.

### 2.3. Study Setting

The study setting is the data collection site [26]. The Vhembe district of the province of Limpopo, South Africa, was the site of the current proposed study. Limpopo, one of South Africa’s nine provinces, is located in the north-eastern part of South Africa. This province is the fifth largest in the country. Limpopo has the lowest percentage of households in informal housing (77,371 or 4.8%) in the country, and more than ten percent of its population (12.9% or 205,432) live in poverty, according to Statistics South Africa [27]. The province of Limpopo is divided into five (5) districts. The researcher selected schools that address child trauma-informed practice in a more rural setting, the Vhembe district and the Dzindi and Mvudi circuit, with limited resources. The region has two main ethnic groups, Tsonga and Venda. All 13 primary schools in the Dzindi and 12 in the Mvudi circuits were included in the research study.

### 2.4. Population

Primary teachers were the target population for possible inclusion. Primary school teachers who participated in the research study taught learners from the ages of 6 to 12 years. Teachers are the people who help learners acquire knowledge and competence, so they can determine if primary school children are facing trauma, hence the need to investigate their understanding concerning child trauma. The total number of primary school teachers within the Mvudi and Dzindi circuits is 518, and in this current study, 26 primary school teachers were included after reaching data saturation. The researcher reached data saturation after interviewing 22 participants but went on and interviewed more primary school teachers to expand the findings.

### 2.5. Procedures

The researchers used purposive sampling to choose the province, district, and circuit for the study. This study is a component of a childhood trauma project already underway in the rural communities of Vhembe district, Dzindi circuit. The researchers felt it was appropriate to conduct the study in this area because of the high prevalence of child abuse in the province and district (multicultural) of different people groups.

All thirteen (13) primary schools in the Dzindi circuit and twelve (12) in the Mvudi circuit, located in the Vhembe district of Limpopo Province, were chosen for this study using total population sampling, as shown in Table 1. The total number of primary school teachers in these circuits is 518. Teachers were selected based on specific inclusion and exclusion criteria to ensure that participants had relevant experience in child development and were in a position to identify childhood trauma in learners.

#### 2.5.1. Inclusion Criteria

- Primary school teachers with at least three years of experience in child development;
- Teachers currently employed at primary schools in the Mvudi and Dzindi circuits;
- Teachers willing to participate in this study and provided informed consent.

#### 2.5.2. Exclusion Criteria

- Teachers not employed in primary schools within the Mvudi and Dzindi circuits;
- Teacher assistants, cleaners, social workers, and gardeners who are not involved in direct classroom teaching.

#### 2.5.3. Recruitment Process

Participants were recruited using purposive sampling, a non-probability sampling technique. The researcher contacted schools within the Mvudi and Dzindi circuits and identified eligible teachers based on the inclusion criteria. The researcher conducted the recruitment process and approached teachers directly in the identified schools.

All teachers from the pool of 518 who met the inclusion criteria were invited to participate in this study. In total, 26 teachers were recruited for this study, with the researcher ensuring that the sample was diverse enough to provide a broad perspective on teachers’ understanding of childhood trauma. It is contended that sample size must be considered when sampling to prevent the disastrous discovery that the sample is too small when data analysis is performed [28]. As a result, based on data saturation, the researchers sampled 26 teachers for this investigation. Data saturation was reached after interviewing 22 participants, but additional teachers were interviewed to expand the findings further.

Data were collected through semi-structured interviews with primary school teachers in the Vhembe District. The researchers personally visited the selected schools and approached the school principals to request permission to conduct the study. After obtaining permission, the principals called all the primary school teachers to a central location, such as the staff room or boardroom, where the researchers explained this study’s purpose, aims, and potential benefits.

The researchers provided detailed information about the study, including its objectives and the voluntary nature of participation. After the explanation, teachers who met the inclusion criteria and were interested in participating in the study volunteered to participate. The informed consent process was conducted in person before the interviews. Teachers who agreed to participate signed the consent form detailing the study’s risks, benefits, and their rights to withdraw without consequences. Teachers who chose not to participate were respectfully excluded from the study. Those who were available and not busy at the time of the meeting stayed for the interview, which was conducted face-to-face immediately after the meeting. For teachers who were occupied with teaching duties or other responsibilities (such as exams), the researcher scheduled interviews at a more convenient time. These face-to-face interviews were conducted at the agreed-upon time. In cases where teachers were unavailable during school hours, the researcher collected their contact numbers and arranged telephone interviews, which were conducted after school hours to accommodate the teachers’ schedules.

The interviews were held in private locations within the school, such as the principal’s office, an empty classroom, or a boardroom (depending on the availability and permission granted by the school). Each interview lasted approximately 30–45 min, and audio recordings were made with the participants’ consent to ensure accurate data collection. The audio recordings were securely stored to maintain confidentiality. Three main questions made up the interview guide were as follows:

1. In your own words, can you please define child trauma?
2. As a primary school teacher, can you identify a trauma-exposed learner? If yes, what are the signs that you are looking for?
3. As a primary school teacher, are you able to manage a trauma-exposed learner? If yes, please elaborate on what you do.

The interviews were flexible, and the researchers took the course the participants recommended. The investigator ensured that the topic was maintained in a non-aggressive manner. Many techniques were used throughout the interviews to promote open communication and enhance thorough explanations of the phenomenon, such as questioning, probing, clarifying, contemplating, and paraphrasing.

### 2.6. Pre-Test

The pretest of the current study was conducted outside of the Dzindi and Mvudi circuits, with three different primary school teachers who were not included as participants in this study. Pre-testing was conducted to ensure the main questions were understandable and unambiguous and to assess the study’s viability [29]. Furthermore, the pre-test showed whether this study could be carried out.

### 2.7. Data Analysis

The data collected through the interviews were analyzed using content analysis, a technique for identifying patterns, categories, and themes within qualitative data. The interview questions explored specific aspects of teachers’ understanding of trauma, including their definitions, identification methods, and suggested interventions. As such, the analysis focused on categorizing the types of responses into key themes such as trauma definitions, signs of trauma, and actions taken to support affected students. These categories allowed for a detailed examination of the data, providing insights into the teachers’ practices and perceptions within the contextual setting of the study. The literature was used to identify and manage the themes. An independent coder was brought in to improve trustworthiness. Agreement between the primary researcher and the independent coder was consistent for most categories. In instances of disagreement, the discrepancies were discussed, and a consensus was achieved through a collaborative review of the data, ensuring accurate categorization and interpretation.

## 3. Results

### 3.1. Demographic Characteristics

Twenty-six primary school teachers from the selected rural communities of the province of Limpopo were interviewed (N = 26). All participants were Black (N = 26). Of these, nine were men and seventeen were women. The teachers’ ages ranged from 20 to over 60 years. The age distribution was as follows: four teachers (15%) were aged 20–29 years, nine teachers (35%) were aged 30–39 years, four teachers (15%) were aged 40–49 years, and nine teachers (35%) were aged 50–59 years. No teachers were over 60 years old. The teachers’ teaching experience ranged from five to over twenty years. The demographic details of the teachers are presented in Table 2.

### 3.2. Themes

This study’s findings are presented using themes and sub-themes from the data analysis (Table 3).

#### 3.2.1. Theme 1: Defining Childhood Trauma

The primary school teachers in this study provided varying definitions of childhood trauma, which mostly centered on issues such as abuse and problems in the home.

##### Sub-Theme 1.1: Comparison with the Formal Definition of Child Trauma

Their definitions aligned in some respects with the formal definition of trauma, as outlined by SAMHSA, which describes trauma as experiences that overwhelm a child’s ability to cope, including abuse, neglect, and witnessing violence. Quotes from participants illustrate this viewpoint:

“*I think childhood trauma is when a child is having problems at home or even at school.*” (Participant No. 1)

Teachers further indicated ways in which trauma can affect children in a school setting:

“*You know, trauma affects so many things, you know, some even commit suicide. You know? It’s not nice and even affects the learners or children’s performances. Family break ups and so on.*” (Participant No. 10)

Although the teachers did not use the formal language from SAMHSA directly, they highlighted key aspects of the definition, such as abuse and family problems, that resonate with the broad understanding of trauma. Their responses focused on how trauma manifests in the child’s environment, often describing it in terms of observable behaviors like changes in concentration and emotional instability.

Primary school teachers associate childhood trauma with household issues. They reported that childhood trauma is when a child has problems at home. Primary school teachers revealed their understanding and stated different circumstances that would occur in the household and cause child trauma. Below is a narrative from one participant:

“*Childhood trauma…we are talking about a child who is having ehm….who is having problems at home or even at school or wherever they can be. Like…ehm…when a child is being abused by parents, grandparents or whoever or when a child is not wanted by the parents or the peer groups the parents or the peer groups do not want a child.*” (Participant No. 2)

Primary school teachers reported that childhood trauma can be in the form of different types of abuse, such as physical abuse, emotional abuse, or sexual abuse. Primary school teachers could not directly define what childhood trauma is but gave examples of the causes of child trauma. Below are some narratives from the participants:

“*Childhood trauma…can be physical abuse, emotional abuse or sexual abuse.*” (Participant No. 2)

“*Some traumas can also be like being raped without you even knowing that, Oh, this is not an OK activity to be happening.*” (Participant No. 8)

#### 3.2.2. Theme 2: Identifying a Child at Risk

Primary school teachers reported different skills in identifying at-risk learners in the classroom. They revealed that a change in behavior, performance, and emotional expression would be indicators, such as a classroom learner crying for no reason, not playing with others, and not concentrating in class. Below are the narratives of the participants:

“*Yes. I can identify him/her just because he cannot concentrate on what I am doing in the classroom.*” (Participant No. 2)

“*At primary, when we teach…we always move around the leaner, and I know…I know in my class; I know my learners, and their performance and their behavior and their performance [sic]. So, if she is dropping down, I will notice that there is something wrong or if there is a behavioral change. I will notice that there is something wrong. Like if she is no longer playing, she is no longer active, eh… the performance is going down, is dropping. I will…sometimes she can, when you are teaching, she can be sleeping in class.*” (Participant No. 12)

##### Sub-Theme 2.1: Behavioral Indicators

Primary school teachers identified childhood trauma through changes in behavior, such as withdrawal, aggression, and lack of concentration. Here are the narratives of the participants:

“*The symptoms are if they are bubbly, they will start to be not…They will come to class and then keep quiet.*” (Participant No. 6)

“*There are kids who you can see that they are being abused. You see that when a child reacts… when a child reacts badly. Then you find them beating others, bullying others, sometimes physically or emotionally. You can see that this child is releasing the anger that they have.*” (Participant No. 3)

##### Sub-Theme 2.2: Emotional Expression

Primary school teachers reported that traumatized children or trauma-exposed learners often express emotions such as sadness, fear, and anger, making it easier for teachers to notice. Participants verbalized the following:

“*They will start crying…regularly crying. Then you would see that…this thing didn’t have to trigger them to cry like this.*” (Participant No. 6)

“*I saw in class that most of the time when I am teaching, when I move close to the child and explain a particular concept, the child will give you space like he fears something. And here at our school, we do not beat children; the law doesn’t allow us to beat children. So, you could see that this child is like he is being abused. When you know what is going on, he will be scared and say, ‘No, I’m just opening the way for you,’ and I said, ‘Why?’ That’s when I saw that there is a problem.*” (Participant No. 4)

##### Sub-Theme 2.3: Changes in Academic Performance

According to primary school teachers, evaluating a learner’s performance in class can reveal whether or not they have experienced trauma. They disclosed that if learners were intelligent, it would be evident that they were traumatized because of the decline in their performance. Below are narratives from a participant:

“*What we are coming across here is that if a child has encountered a scary situation, they do not want to sit even in class or write. Sometimes, they don’t even want to come to school. As I mentioned before, there was a child who saw his siblings’ dead bodies hanging. It affected that child so badly. His school performance dropped, and he didn’t even want to attend school.*” (Participant No. 9)

#### 3.2.3. Theme 3: Managing a Child at Risk

Primary school teachers reported that they would attempt to talk to learners and may report to senior staff, involve parents, or refer to external resources. Two sub-themes emerged: building rapport with the learner and referral to the senior/principal. Below is a narrative from the primary school teacher on how they would handle or manage a trauma-exposed learner.

##### Sub-Theme 3.1: Building Rapport with the Learner

Teachers attempt to talk to learners and may report to senior staff or involve parents. Primary school teachers reported that they aim to connect with learners to understand their issues. Quotes from participants illustrate this viewpoint:

“*I will call him/her and ask her to come to the office and ask him or her about the problem she’s having.*” (Participant No. 1)

“*It was so difficult because she didn’t want to say anything. She would say ‘I’m OK. I’m OK. I’m OK’. Until I was like, let me just be friends with her. Let me just take her closer to me.*” (Participant No. 25)

“*In our classroom, we have some small rooms called storerooms where we put our things. Then, I will call the learner into that small room, being two. Then, I will ask her a few questions before I ask her a few questions. I will talk to her nicely as a friend, and I will also show that learner love so that she will be free to tell me what is wrong or what happened. Then, I will ask her eeehh… a few questions. I can say, “Since last week, my child, it seems you’re not well. Do you have anything, or do you have any problem, or is there something wrong?” Then, if she is not answering well, maybe she is afraid. I will…eh… talk to her, showing her love, things like that so that she will try to answer*” (Participant No. 12)

##### Sub-Theme 3.2: Referral to Senior/Principal

Teachers reported that when they come across a trauma-exposed learner, they refer the learner to a senior teacher or take the matter forward to the principal. Below is one narrative from a participant:

“*You find that the teachers know that the child has a problem, then we take the child to the Head of Department (HOD), then from HOD to the principal, then they will call the social workers.*” (Participant No. 11)

Additionally, when the researcher probed further if they have school-based social workers, a participant responded with the following:

“*No, we don’t have. And if you can check, our school is too big; we have more than a thousand, nearly one thousand six hundred primary school learners. We did share with the principal that there is a need, but we don’t have one yet.*” (Participant No. 2)

“*Yes. So, we report to our seniors. It’s either the HOD who is our senior or the principal.*” (Participant No. 4)

## 4. Discussion

The effects of childhood trauma on learners’ academic performance, social connections, and emotional well-being provide serious issues for educational environments. As first responders and caretakers, primary school teachers are essential in spotting and helping traumatized children. The presented discussion provided a summary of this study’s results, emphasizing significant issues such as the lack of consensus on the definition of trauma, techniques for identifying at-risk children, and the handling of kids who have experienced trauma. It also highlighted critical gaps that must be filled to improve teachers’ ability to assist trauma-exposed learners.

Trauma-exposed primary school learners need trauma-informed teachers who will be able to help them. In the current study, primary school teachers revealed different perspectives on child trauma, and they frequently linked it to problems like abuse, dysfunctional families, or other adverse events. The abovementioned diversity highlights the necessity of consistent training programs and policies to guarantee a shared understanding among primary school teachers. Teachers could find it challenging to recognize trauma in various circumstances and respond to it consistently if there are unclear definitions and frameworks in place [30,31]. For example, a few primary school teachers only paid attention to obvious signs of abuse or neglect, while others ignored less obvious signals of psychological trauma or mental distress. Standardizing criteria through extensive training programs could improve early intervention and support mechanisms by giving teachers the knowledge and sensitivity to identify trauma in its different forms.

Primary school teachers primarily identify trauma-exposed learners by using behavioral, emotional, and academic indicators to spot trauma-exposed learners. Behavior changes that cause teachers to look into a learner’s case more often include anger, withdrawal, and sudden mood changes. Emotional manifestations like grief, worry, or anxiety are also essential markers since they frequently represent underlying trauma that kids might not express out loud [32]. This study did, however, draw attention to the fact that teachers were able to identify clear, observable symptoms of trauma, such as changes in behavior (e.g., aggression or withdrawal), emotional expression (e.g., crying or fear), and academic performance. However, less obvious symptoms, such as internalized emotions like anxiety, somatic complaints (e.g., headaches or stomachaches), or subtle behavior changes, were not explicitly mentioned by teachers in this study, which can result in under-identification and a delay in receiving help. It takes continual professional development and access to resources that expand their knowledge of trauma-informed techniques for teachers to recognize and interpret these indicators [33] and to understand the management of childhood trauma.

The findings of the current study highlighted a significant gap in the understanding and management of child trauma among primary school teachers. Many teachers expressed uncertainty and relied heavily on referring trauma-exposed learners to school principals or heads of department (HODs), indicating a need for more confidence in handling such complex issues themselves. This raises pertinent questions about the preparedness and training of school leadership, such as principals, in trauma-informed practices. Without adequate training or resources within the school setting, the effectiveness of these referrals may be compromised. Moreover, the reliance on external social workers, as noted by teachers who reported outsourcing support services, underscores the limited availability of in-house mental health professionals. This systemic reliance on external resources highlights the need for comprehensive, school-based support systems that integrate trauma-informed practices into everyday school operations. Future efforts should focus on enhancing the capacity of school leaders and educators alike to effectively identify, support, and advocate for trauma-exposed learners within the school environment.

While educators are committed to supporting learners who have experienced trauma, their effectiveness is often limited by a lack of resources and training. Teacher training programs must include trauma-informed practices to enhance teachers’ capacity to recognize, handle, and steer clear of trauma-related issues in schools. These include using trauma-sensitive classroom management strategies, being conscious of how trauma affects the nervous system, and taking care of oneself to avoid burnout.

The findings of this study highlighted several critical gaps in the identification and management of childhood trauma in primary schools. One significant gap is the lack of a standardized definition of trauma among teachers, which leads to inconsistent recognition and response to trauma-exposed learners. Teachers often rely on their interpretations of trauma, which can result in some cases being overlooked or misdiagnosed. Another critical gap is the insufficient ongoing professional development and training in trauma-informed practices for teachers. Without adequate training, teachers may struggle to identify trauma and provide appropriate support, limiting early intervention and exacerbating the impact on students. Furthermore, the preparedness of school leadership, including principals and heads of departments, is another critical gap. Many teachers reported relying heavily on referrals to senior staff, indicating that school leaders may lack the necessary trauma-informed expertise to manage cases effectively. Additionally, the lack of in-house mental health professionals within schools is a significant gap, as schools often depend on external social workers or community resources, which can be limited and may delay timely intervention. This issue is compounded by disparities in access to mental health services, particularly in underprivileged and rural areas where resources are scarce. Addressing these gaps requires systemic changes, including comprehensive trauma-informed training for teachers and school leaders, the integration of in-house mental health support services, and policy changes to ensure equitable access to mental health resources for all children. By incorporating trauma-informed teaching practices into professional development courses, teachers may establish classrooms that foster empathy, resilience, and inclusive education for all kids [2].

Moreover, to effectively address the complex needs of traumatized adolescents, collaboration between schools and other support systems, such as social workers or community health agencies, is imperative [34,35]. Disparities in access to mental health treatments and resources for assistance, however, continue to exist, especially in underprivileged groups and rural locations [36]. Encouraging fair access to high-quality healthcare necessitates forging strategic alliances, pushing for legislative changes, and making focused investments in community-based support systems. Schools can improve their ability to offer prompt treatments, encourage constructive coping mechanisms, and aid in the rehabilitation of kids facing trauma-related issues by fortifying these cooperative endeavors.

## 5. Limitations of the Study

This study had several limitations. Social desirability bias and response bias may have influenced teachers’ responses, as they could have been motivated to provide answers they felt were expected or socially acceptable, particularly when discussing sensitive topics like trauma, despite being assured they could speak freely in a private setting. Additionally, this study focused on teachers from the Dzindi and Mvudi circuits in Limpopo province, which limits the generalizability of the findings to other regions. While data saturation was reached with 26 participants, a larger sample size could enhance the robustness of the results. Future research should consider broader geographic and participant diversity to confirm and expand upon these findings.

## 6. Conclusions

This study highlighted the importance of trauma-informed training for primary school teachers. While teachers demonstrated familiarity with specific traumatic events, the results did not indicate a consistent or comprehensive understanding of child trauma across all participants. Instead, the teachers primarily identified trauma in terms of observable behaviors such as aggression, withdrawal, and academic difficulties. This suggests that while teachers can identify some signs of trauma, their understanding may not encompass the full spectrum of trauma’s impact, particularly less visible or internalized symptoms.

To enhance educators’ capacity to support trauma-exposed learners, targeted professional development programs are essential. These programs should not only focus on identifying visible signs of trauma but also equip teachers with the skills to recognize and respond to less apparent symptoms, such as emotional distress or somatic complaints. Furthermore, the need for further research on effective trauma-informed practices in schools is evident. Incorporating evidence-based strategies into teacher training and school policies is crucial for fostering a supportive learning environment for all students.

Additionally, this study underscored the importance of building partnerships between schools, mental health professionals, and community resources to ensure comprehensive support for traumatized learners. Advocacy for policies prioritizing trauma-informed practices within educational settings is essential to provide consistent and equitable support for all students. Through these efforts, we can create an educational environment that not only acknowledges the impact of trauma but is also equipped to manage it effectively, thereby enabling every learner to thrive.

## Figures and Tables

**Table 1:** Primary schools in the Dzindi and Mvudi circuits.

Primary Schools in the Dzindi Circuit	Primary Schools in Mvudi Circuit
Makumbane primary school	Lukunde primary school
Dambalwashe primary school	Tshedza comprehensive school
Mapate primary school	Lukhaimane primary school
Mahematshena primary school	Mafenya primary school
Belemu primary school	Maungani primary school
Tshivhale primary school	Matsika primary school
Tshivhambe primary school	Mbahe primary school
Muthundinne primary school	Nweli primary school
Tshiwedza primary school	Tshishonga primary school
Manamani primary school	Tshiluvhi primary school
Dzwerani primary school	Mulamuleli primary school
Tshisaulu primary school	Tshamavhudzi primary school
Mabuduga primary school	

**Table 2:** Demographic characteristics.

Item	Attributes	Frequency	Percentage
Age	20–29	4	15%
	30–39	9	35%
	40–49	4	15%
	50–59	9	35%
	≥60	-	-
Gender	Males	09	35%
	Females	17	65%
Years of experience as a teacher	5–10	15	58%
	10–15	4	15%
	15–20	3	12%
	>20	4	15%
The age range of learners taught	5–7 years	4	15%
	8–10 years	9	35%
	10–12	13	50%

**Table 3:** Themes and sub-themes.

Themes	Sub-Themes
Defining childhood trauma	Comparison with the formal definition of child trauma.
Identifying a child at risk	Behavioral indicators
	Emotional expression
	Changes in academic performance
Managing a child at risk	Building rapport with the learner
	Referral to the seniors

## Data Availability

All the data sets used for the study are available from the corresponding author upon reasonable request due to privacy concerns regarding personal information and ethical considerations in ensuring participant confidentiality.
